# Mortality in people with dementia, delirium, and unspecified cognitive impairment in the general hospital: prospective cohort study of 6,724 patients with 2 years follow-up

**DOI:** 10.2147/CLEP.S174807

**Published:** 2018-11-23

**Authors:** Simona Hapca, Bruce Guthrie, Vera Cvoro, Feifei Bu, Alasdair C Rutherford, Emma Reynish, Peter T Donnan

**Affiliations:** 1 Population Health and Genomics, School of Medicine, University of Dundee, Dundee DD2 4BF, UK, s.z.hapca@dundee.ac.uk; 2 NHS Fife, Kirkcaldy, Fife KY2 5AH, UK; 3 Dementia and Ageing Research Group, Faculty of Social Science, University of Stirling, Stirling, FK9 4LA, UK

**Keywords:** acute admission, elderly, cognition, function, non-proportional hazards

## Abstract

**Purpose:**

Cognitive impairment is common in older people admitted to hospital, but the outcomes are generally poorly understood, and previous research has shown inconsistent associations with mortality depending on the type of cognitive impairment examined and duration of follow-up. This study examines mortality in older people with any cognitive impairment during acute hospital admission.

**Patients and methods:**

Prospective cohort of 6,724 people aged ≥65 years with a structured cognitive assessment on acute admission were included in this study. Cognitive spectrum disorder (CSD) was defined as delirium alone, known dementia alone, delirium superimposed on known dementia, or unspecified cognitive impairment. Mortality associated with different types of CSD was examined using a non-proportional hazards model with 2-year follow-up.

**Results:**

On admission, 35.4% of patients had CSD, of which 52.6% died within 2 years. After adjustment for demographics and comorbidity, delirium alone was associated with increased mortality in the 6 months post-admission (HR =1.45, 95% CI 1.28–1.65) and again after 1 year (HR =1.44, 95% CI 1.17–1.77). Patients with known dementia (alone or with superimposed delirium) had increased mortality only after 3 months from admission (HR =1.85, 95% CI 1.56–2.18 and HR =1.80, 95% CI 1.52–2.14) compared with patients with unspecified cognitive impairment after 6 months (HR =1.55, 95% CI 1.21–1.99). Similar but partially attenuated associations were seen after adjustment for functional ability.

**Conclusion:**

Mortality post-admission is high in older people with CSD. Immediate risk is highest in those with delirium, while dementia or unspecified cognitive impairment is associated with medium- to long-term risk. These findings suggest that individuals without dementia who develop delirium are more seriously ill (have required a larger acute insult in order to precipitate delirium) than those with pre-existing brain pathology (dementia). Further research to explain the mortality patterns observed is required in order to translate the findings into clinical care.

## Introduction

The rising prevalence of cognitive impairment driven by rapid population aging is a growing public health concern and presents major challenges to all health services, including hospitals. Cognitive impairment has been reported to be present in 26%^1^ to 47.9%^2^ of hospitalized older people, with varying prevalence depending on the population studied (eg, specialist settings vs unselected medical admissions, age range) and the assessment methods used.

Cognitive impairment in hospital inpatients can be due to a number of overlapping conditions.^3^,^4^ People may have known dementia before admission, may develop delirium due to an acute illness precipitating admission, may have delirium superimposed on known dementia, or may have unspecified cognitive impairment defined as impaired cognition without a formal diagnosis of dementia or delirium. We have recently proposed the term “cognitive spectrum disorder” (CSD) to include any of these forms of cognitive impairment.^5^ Older people admitted to hospital with a CSD are a heterogeneous and highly vulnerable population. Due to overlapping symptoms, differentiation between the different types of CSD in clinical settings can be difficult. In general it is known that they have poor outcomes in terms of longer hospital stay^6^,^7^ and higher associated costs.^8^

People with various individual CSDs have been shown to have a high mortality in many studies internationally.^9^–^15^ A US population study reported that 39.6% of older people with dementia had died by the end of 6.5 years follow-up, compared to 18.5% of people without dementia.^10^ Mortality rates are particularly high in hospitalized people with CSD.^2^,^16^–^19^ Sampson et al estimated a 24% in-hospital mortality rate in people with dementia aged ≥70 years,^2^ and Fick et al reported 25% mortality within a month of admission in older people with delirium superimposed on dementia.^17^ A recent study investigating mortality of hospitalized older people in Brazil found an in-hospital mortality rate of 32% in people with delirium superimposed on dementia on admission and 29% in people with delirium alone, as opposed to 12% in people with dementia alone and 8% in those without delirium or dementia.^19^ For those surviving to discharge, mortality in the subsequent year in those with dementia was significantly higher than that in those with delirium alone (43.9% vs 36.2%). This evidence suggests that post-admission mortality risk in patients with CSD may vary over time depending on the underlying condition. However, most of the previous research studies have examined only some CSD conditions, using relatively small cohorts of selected volunteers in specialist geriatric medicine settings with variable lengths of follow-up. It is difficult to be sure whether the varying findings of different studies reflect true differences in mortality in different settings or simply patient selection and analytical choices.

We can hypothesize that patients with any CSD are at increased risk of death and that associated physical illness severity (the precipitant of delirium) results in increased early risk of death. In addition, the presence of CSD in older people is strongly correlated with low functional ability, which in itself is associated with poor outcomes following hospital admission.^16^,^19^,^20^ However, whether the CSD associated functional impairment can fully explain the poor outcomes in people with CSD admitted to hospital is not well understood.

### Aim

The aim of this study was to use a large population-based data set of older people admitted to hospital to examine the mortality risks associated with different types of CSD up to 2 years after hospital admission adjusted for known confounders including patients’ functional ability.

## Materials and methods

### Population

This is a prospective cohort study of people aged ≥65 years admitted as a medical emergency between January 1, 2012 and December 31, 2013, with 2-year follow-up, using linked national data.

NHS Fife provides care to a varied urban and rural population of ~360,000. During the study, all emergency medical admissions were via a single acute medical unit (AMU) in a 640-bedded district general hospital. Trained specialist nurses assessed people aged 65+ years on admission using a locally developed Older Persons Routine Acute Assessment (OPRAA) based on the principles of comprehensive geriatric assessment.^21^ OPRAA was completed in the first 24 hours of admission, and by design, individuals expected to die because terminally ill or transferred to critical care or with a predicted length of stay <24 hours were not assessed.

An incident cohort was defined as those aged 65+ years who had received an OPRAA assessment during the 2-year study period and had no previous acute medical admission in the prior 6 months. The incident cohort aimed to identify individuals at the beginning of a new interaction with acute hospital services and follow them through their subsequent care capturing all re-admissions and mortality. We used clinical judgment to define this, with a consensus that a patient with no admissions in the previous 6 months was starting a new interaction.

### Data and covariates

Data for all eligible incident admissions were identified from the Scottish Morbidity Records 01 (SMR01) data, which is a validated NHS Scotland routine data set recording admission and discharge dates and destinations, and discharge diagnosis using ICD-10 codes. Discharge diagnosis (excluding dementia) from all previous admissions was used to calculate each participant’s Charlson Comorbidity Index (CCI) on admission.^22^

The OPRAA data set was used to identify patients with an OPRAA assessment completed, which recorded CSD (with the following groups: delirium alone, dementia alone, delirium superimposed on known dementia, and unspecified cognitive impairment in the absence of delirium and/or dementia; Box 1) and functional status based on assessment of activities of daily living (ADL with the following groups: persistently low ADL and changed ADL; Box 1).

Data on all community dispensed prescriptions were used to create an additional multimorbidity score, calculated as the number of drugs (defined as number of distinct British National Formulary subsections) prescribed to the patient 84 days prior to admission.^23^

Box 1 **Definitions of cognitive spectrum disorder and categorization of functional status**

**Definitions of cognitive spectrum disorder**

Delirium was defined as a clinical diagnosis of delirium made by the trained specialist nurse completing the OPRAA.^8^ OPRAA included administration of the Confusion Assessment Method (CAM) using the original (pre-2014) recommended scoring^24^ which was subsequently revised to address low sensitivity in clinical applications, so for the purposes of this analysis we used the overall clinical assessment made by the trained nurses.

Known dementia was defined as documentation during the OPRAA assessment of the presence of a preadmission diagnosis of dementia from self/informant report and/or hospital and primary care records; OR a prior ICD-10 code for dementia recorded during an acute hospital (SMR01) or psychiatric admission (SMR04); OR prior community prescribing of a drug for dementia (anticholinesterase inhibitors or memantine as listed in British National Formulary, chapter 4.11).

Delirium superimposed on dementia was defined as the presence of delirium in a patient with known dementia.

Unspecified cognitive impairment was defined as an Abbreviated Mental Test (AMT) score <8 in people with no delirium and no known dementia.^8^

**Categorization of functional status**

Functional status was assessed during OPRAA using the activities of daily living (ADL) assessment of six basic activities: eating, bathing, dressing, toileting, transferring (walking), and continence adding up to a maximum score of 6.^25^ Based on patient and/or informant report, functional status was assessed at 12 weeks before admission (pre-ADL) and on admission (current-ADL) based on direct observation. Participants were then defined as having:

- Persistently low-ADL (pre-ADL score <5 all of whom had current-ADL <5)
- Changed-ADL group (pre-ADL score ≥5 and current-ADL score <5)
- Persistently high-ADL (both pre- and current-ADL scores ≥5).

**Abbreviation:** OPRAA, Older Persons Routine Acute Assessment.

All patients were followed up from the date of their index admission to the earliest of their date of death or 2 years follow-up. Death was ascertained from the Community Health Index data set (CHI – the NHS Scotland population register), which was also used to define participant age, sex, and postcode-defined socioeconomic status (measured using quintiles of the Scottish Index of Multiple Deprivation) on admission.^26^

The CHI number (the NHS Scotland unique patient identifier) was used to deterministically link SMR01 to CHI, OPRAA, SMR04, and community dispensed prescribing.

### Missing data

Data on delirium diagnosis was missing in 3.7% of cases in the incident cohort. Based on OPRAA alone, 9.8% of cases were recorded as having known dementia and 20.3% of cases had missing data for dementia. After adding information on dementia from SMR01, SMR04, and prescribing data sets, the percentage of people with known dementia was 15.3%, with the rest of cases being treated as absence of dementia. 20.9% of cases had a missing AMT score within OPRAA, of which 15.5% had neither delirium nor dementia; these were classified as not having any CSD. 27% of ADL scores within OPRAA had missing values. Multiple imputation based on PROC MI in SAS was used to impute the missing ADL categories based on five imputations and assuming data were missing at random.^27^ The imputation model for missing ADL status was based on the logistic regression and included all the variables used in the survival model (sex, age, deprivation status, residential status, comorbidity, and number of drugs 84 days prior to admission), as well as outcome variables that were thought to be predictive of the missing ADL values such as length of hospital stay, mortality at 1 month, 3 months, 6 months, 1 year, and 2 years. A pooled analysis of the five imputed data sets using PROC MIANALYSE in SAS was performed as main analysis, and a sensitivity analysis was carried out on the complete ADL cases.

### Statistical analysis

Summary statistics based on numbers and the corresponding proportions for categorical variables and mean with standard deviations for continuous variables were used to describe prevalence of the different CSDs in older people admitted to AMU and how this varied with their demographics. Characteristics of older people in the CSD groups were examined in terms of CCI (with groups: zero, one, two to five and six and over), number of drugs 84 days prior to admission (with groups: zero drugs, one to five drugs, six to ten drugs, and eleven and more), and ADL function (persistently low-ADL, changed-ADL group, or persistently high-ADL; Box 1).

Analysis of time to death with a 2-year follow-up from admission was initially assessed with Kaplan–Meier survival plots with the corresponding mortality rates. Cox proportional hazards models using PROC PHREG in SAS were initially used to investigate the association of CSDs and survival. Assessment of the proportional hazards assumption, derived from the cumulative sum of martingale residuals and Kolmogorov-type supremum test using the ASSESS statement under PROC PHREG,^28^ showed that some of Cox model covariates did not meet this assumption, so a non-proportional hazards model with time-varying coefficients was fitted.^29^ Time-varying coefficients were modeled based on a piecewise constant model function, where the 2-year follow-up was initially split into five time intervals: up to 1 month (implemented as up to 30 days), 1–3 months (31–90 days), 3–6 months (91–180 days), 6 months to 1 year (181–365 days), and 1–2 years (366–730 days). The Akaike’s Information Criteria (AIC) was used to optimally choose the time points (among 30, 90, 180, and 365 days) when a change in HR was supported by the data, if no change in HR between consecutive time periods was supported by the data, then these periods were re-grouped and an HR was calculated for the re-grouped time period. The effect of CSD on survival was estimated in terms of unadjusted HRs (unadjusted model a) and HRs adjusted for demographics and comorbidity variables (adjusted model b). Additionally, HRs adjusted further for ADL functional status (adjusted+ ADL model c) were calculated to specifically determine how much of the increase in hazard ratio in people with CSDs was explained by their functional status. The AIC was used for variable selection in the adjusted models b and c.

Finally, to test for a difference in mortality risks between delirium superimposed on dementia and delirium alone, delirium superimposed on dementia and dementia alone, and unspecified cognitive impairment and dementia alone, the non-proportional hazards model was repeatedly fitted by changing the reference category with the different CSD categories instead of the No-CSD category.

Data analysis was carried out using SAS^®^ 9.4 (SAS Institute Inc., Cary, NC, USA).

### Ethical approval

Data provision and initial management including linkage was carried by the University of Dundee Health Informatics Centre (HIC, https://www.dundee.ac.uk/hic), with analysis of anonymized data carried out in an ISO27001 and Scottish Government accredited secure safe haven. HIC Standard Operating Procedures have been reviewed and approved by the NHS East of Scotland Research Ethics Service, and consent for this study was obtained from the NHS Fife Cal-dicott Guardian.^38^

## Results

### Description of the cohort

Between January 2012 and December 2013, there were 17,151 admissions of patients aged ≥65 years to the AMU. About 9,331 of these admissions were incident admissions, of which 6,724 (72%) had an OPRAA. The mean age for patients in the incident OPRAA cohort was 79.2 years, 56.3% were women, and 7.4% were admitted from a care home. About 20.5% of patients lived in the most deprived fifth of areas, whereas 14.6% lived in the most affluent fifth.

CSD was present in 35.4% of the incident OPRAA admissions. Delirium alone was present in 15.8%, known dementia alone in 7.8%, delirium superimposed on dementia in 7.6%, and unspecified cognitive impairment in 4.2% of admissions.

People with CSD were older than those without (Table 1, mean age 82.1 vs 77.6 years) and 59.2% of patients with CSD were women vs 54.6% of those without. About 17.9% of people with CSD were admitted from a care home vs only 1.7% of those without with 29.9% of people with dementia alone and 34.1% of people with delirium superimposed on dementia residing in a care home (Table S1). We considered all these differences large enough to potentially influence any observed association between CSDs and mortality. There were no major differences by socioeconomic deprivation.

In general, the presence of any CSD was strongly associated with low functional ability with 81.0% of patients with CSD having a persistently low-ADL or changed-ADL, compared to 41.8% of patients without a CSD patterns of ADL varied by CSD, with over 50% of patients with known dementia having a persistently low-ADL, whereas almost 50% of patients admitted with delirium alone had a changed-ADL at admission (Table S1).

### Survival analysis

**Figure 1:**
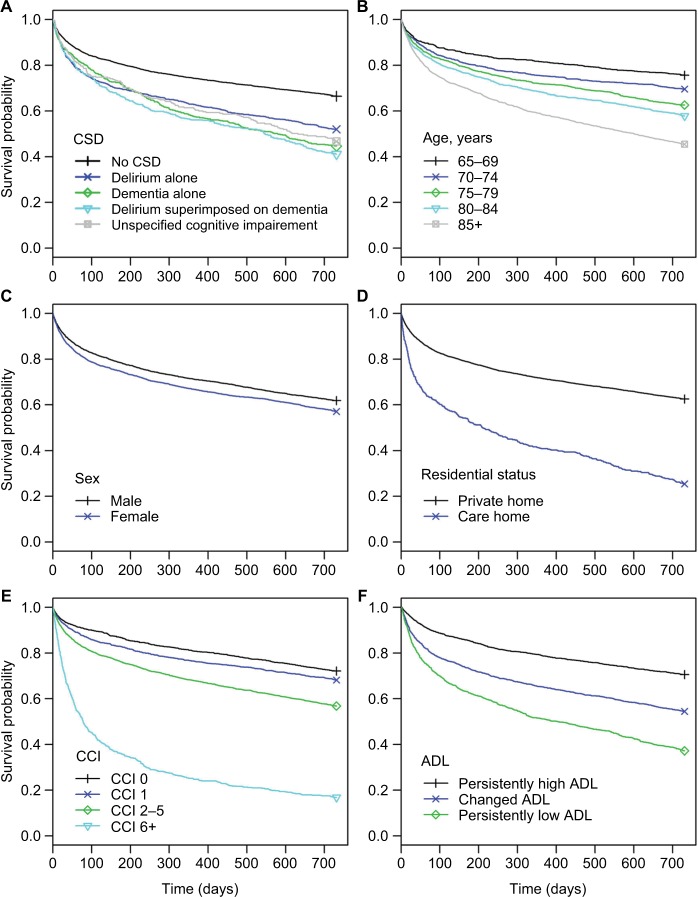
Kaplan–Meier survival functions for the CSD groups (**A**), age groups (**B**), sex (**C**), residential status (**D**), CCI groups (**E**), and ADL functional status (**F**). **Abbreviations:** CSD, cognitive spectrum disorder; CCI, Charlson Comorbidity Index; ADL, activity of daily living.

Kaplan–Meier survival curves for the different subgroups of patients and the associated mortality rates are presented in Figure 1 and Table S2. Mortality was higher in patients with CSD with 52.6% dying within the 2-year follow-up compared to 33.5% of those without (Figure 1A). Increasing age was associated with lower survival (Figure 1B), as was sex, with men being at higher risk (Figure 1C). There was poorer survival for people admitted from a care home than private home (Figure 1D, 74.6% vs 37.5% 2-year mortality rate) and people with a high comorbidity index (CCI 6+) also had a very poor survival (Figure 1E, 83.2% 2-year mortality rate). Survival in patients with persistently low-ADL was generally poor with 62.9% of this group of patients dying within the 2-year follow-up time, the corresponding figures for those with a changed-ADL and persistently high-ADL being 45.6% and 28.4% (Figure 1F). The results of the Cox proportional hazards model are shown in Table S3. However, the assumption of proportional hazards over time was violated for several variables indicating that the Cox model was misspecified.

**Figure 2:**
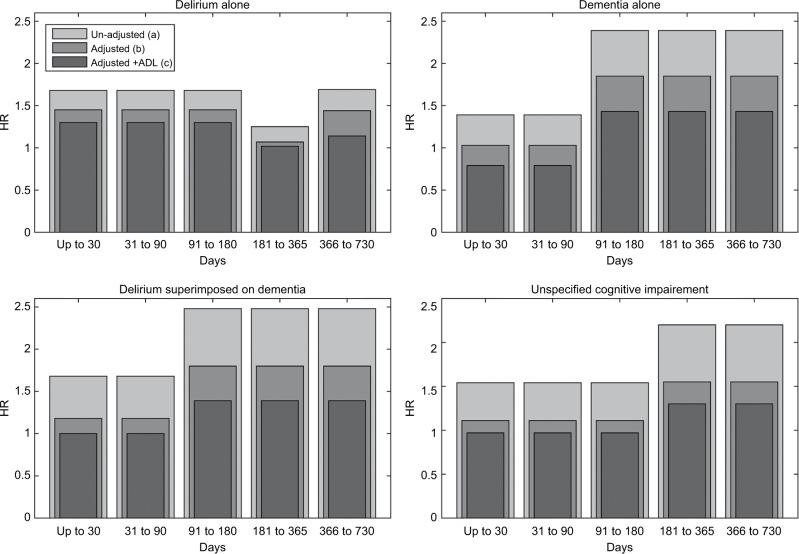
Changes in hazard ratio estimates over time unadjusted and adjusted for demographics, comorbidity variables (adjusted), and ADL functional status (adjusted + ADL), for the different types of CSD. **Abbreviations:** CSD, cognitive spectrum disorder; ADL, activity of daily living.

The non-proportional hazards model results showing changes over time in the HR estimates associated with the different types of CSD are illustrated in Figure 2. Unadjusted HR estimates of the non-proportional hazards model (Table 2, model a) showed that, compared to patients without CSD, patients with delirium alone had a higher risk of death in the first 6 months from admission and again after 1 year, whereas risk of death in patients with dementia (alone or with delirium superimposed) was increased in the first 3 months and further increased over longer follow-up. For patients with unspecified cognitive impairment, the risk of death was increased throughout follow-up compared to those without CSD. All other modeled variables apart from the number of drugs showed significant associations with mortality in all or most time periods.

After adjustment for demographics and comorbidity, similar patterns of mortality risk over time persisted for people with CSD (Table 2, model b). Patients with delirium alone were at an increased risk of death compared to those without CSD in the first 6 months post-admission (HR 1.45, 95% CI 1.28–1.65) and between 1 and 2 years after admission (HR 1.44, 95% CI 1.17–1.77). Patients with dementia (with or without superimposed delirium) were at an increased risk after 3 months from admission (HR 1.85, 95% CI 1.56–2.18 and HR 1.80, 95% CI 1.52–2.14 respectively), and patients with unspecified cognitive impairment had an increased risk of death only after 6 months from admission (HR 1.55, 95% CI 1.21–1.99).

Patients’ sex, age, care home residence, and CCI were significantly associated with mortality with non-constant HRs providing a better fit over the 2-year follow-up (Table S4, model b). In the adjusted model, associations with the numbers of drugs dispensed were weaker and less consistent, and social deprivation was found not significant and removed from the model.

Patients with persistently low-ADL and changed-ADL were at higher risk of death in the first month following admission compared to those with persistently high-ADL (Table S4, model c, persistently low-ADL HR 2.26, 95% CI 1.74–2.94 and changed-ADL HR 2.31 95% CI 1.81–2.96). These associations weakened in the period 1 month to 2 years (persistently low-ADL HR 1.73, 95% CI 1.52–1.96 and changed-ADL HR 1.28 95% CI 1.13–1.47). Reflecting the strong correlation between the presence of CSD and functional decline (Table 1), adjustment by ADL status attenuated the associations between CSD and mortality; however, mortality risk in patients with CSD remained high showing similar temporal patterns to those before adjustment for ADL (Table S4, model c).

The sensitivity analysis conducted on the complete cases to account for the effect of missing ADL showed a general agreement between the survival models performed on the imputed data (Table S4, model c) and the complete cases data (Table S5). The HR estimates obtained from the model fitted to the imputed data are comparable to the HR estimates obtained from the complete cases data with the exception of HR estimates for patients with delirium after 1 year from admission. When applied to the complete cases only, the non-proportional survival model adjusted for ADL indicated that patients admitted with delirium are not at an increased risk of death after 6 months from admission until the 2-year end of follow-up time (HR =1.14, 95% CI =0.90–1.45), whereas based on the imputed data set, this group of patients are still at risk between the 1 year and 2 years follow-up time (HR =1.27, 95% CI =1.11–1.57), a result which is consistent with the results of the unadjusted model, or the model adjusted for demographics and comorbidity only (models a and b in Table 2).

### Comparing mortality risks among the different types of CSD

HR estimates comparing mortality among the different CSD groups are presented in Table S6. The survival model adjusted for demographic characteristics, residence status, and comorbidities showed that patients who were admitted with delirium alone had an increased risk of death in the first 3 months following admission than patients with delirium superimposed on dementia (HR =1.30, 95% CI 1.06–1.59), the situation being completely reversed after 3 months in these two groups of patients (HR =0.72, 95% CI 0.59–0.86). In turn, mortality risk associated with delirium superimposed on dementia was not different from dementia alone for the whole follow-up time (HR =1.05, 95% CI 0.84–1.23). Moreover, patients admitted with an unspecified form of cognitive impairment had a reduced risk of death compared to patients with known dementia alone in the period between 3 and 6 months from admission (HR =0.60, 95% CI 0.36–0.99).

## Discussion

In this study, over a third of people aged ≥65 years with an incident admission to the AMU had a CSD, of which more than half died in the 2 years after admission. People with CSD at the time of admission had a higher mortality than people without CSD, which is not explained by differences in age, sex, comorbidity, care home residence, or functional status. The study found that the risk of death was not constant over the 2-year follow-up time but varied with the underlying CSD condition; compared to people without CSD, those with delirium have an increased risk of death in the short term following admission, while people admitted with delirium superimposed on dementia, dementia alone, or unspecified cognitive impairment have increased medium- to longer-term risk.

These findings are consistent with Inouye’s concept that delirium occurs in the face of both precipitating and predisposing factors.^30^ We can postulate that patients with delirium alone are on average more severely ill from precipitating factors such as a physical illness causing the delirium resulting in increased early risk of death. Those with delirium superimposed with dementia have a worse 2-year prognosis overall in this study (Figure 1A), but after adjustment for baseline variables, their mortality is similar to that of patients with dementia alone. We can postulate that their predisposition to delirium as a result of existing dementia means that they require a smaller insult from physical illness (ie, less severe physical illness) to result in delirium, and their mortality remains that of their underlying dementia, comorbidity, and functional status.

### Strengths and limitations

An important strength of the study is the examination of all types of CSD in a large, unselected population of people aged ≥65 years admitted to hospital as a medical emergency who received a structured cognitive assessment on admission, and with complete 2-year follow-up of mortality after admission using linked data from routine clinical practice. The study included 6,724 incident patients with a new episode of acute hospital care, which is more than the total patients in all studies included in the most recent systematic reviews of dementia^31^ and delirium^32^ in hospital inpatients. The study is novel in its use of a non-proportional hazards survival model to account for the widespread violation of the proportional hazards assumption. Although we do not know whether previous studies used models that violated this assumption because they do not discuss model assumptions, one explanation for inconsistencies in findings is that averaging hazards that are changing over time may lead to misleading findings (see Table S3). The patients in our study had a long post-admission follow-up time; however, the use of the non-proportional hazards model allowed appropriate estimation of short- and long-term risk of mortality associated with the different type of CSD, information that is critical to health carers to appropriately manage this group of inpatients.

The main limitation is that all admissions were based within the same health care system, which may limit generalizability although this is true of most previous studies. In addition, by design OPRAA assessment was not offered to patients with brief admissions or patients who were critically ill, meaning that only 72% of incident admissions in the study period were included. Again though, this is similar to most consented research cohorts which often exclude patients who are admitted for <24 hours and which have similar proportions of all admissions included.^2^ Of those patients who did not undergo an OPRAA assessment (n=2,600), 1,788 (68.8%) had a length of stay ≤2 days, 177 (6.8%) died within 1 month, and 758 (29.2%) died within 2 years. Mortality in the group of patients who did not undergo an OPRAA assessment is slightly lower compared to the OPRAA group; however, given the fact that the large majority of patients underwent an OPRAA assessment, the impact of those who did not undergo an OPRAA assessment on prevalence and outcomes will not be strong. Additionally, 27% of OPRAA patients had missing data for functional status. Multiple imputation was used to impute the missing values assuming data were missing at random. Missingness in the ADL may be related to a short length of stay or patients being in a critical state during admission resulting in a poor outcome, in which case the missing at random assumption may not be valid. However, the complete case sensitivity analysis showed good agreement with the main analysis. Another limitation of the study relates to the fact that information on the marital status of the patient or living arrangements was not available in this study. These characteristics might act as potential confounding, although evidence from previous studies is mixed regarding the associations between these factors and mortality.^16^,^19^ Like all observational data analyses, we cannot rule out residual confounding as an explanation for the observed results.

Other limitations of the OPRAA cohort^5^ arise from: 1) accuracy of brief assessment tools, 2) cross-sectional nature of assessment, and 3) lack of full dementia diagnostic workup. The OPRAA assessment used relatively simple instruments suitable for identifying delirium and cognitive impairment in a routine clinical context which may not always match assessment using gold-standard research instruments but do reflect real-world practice.

### Comparison to other studies

A number of previous studies have examined mortality in hospitalized people with CSD. Some studies on delirium^16.20,33^ have reported significant associations between delirium and mortality at 1 year, whereas others have identified significant associations with short-term mortality^19^,^34^ but not at 1 year.^35^,^36^ Evidence is also mixed regarding people with dementia, with some studies reporting short-2 or long-term^19^ increased mortality, while others found no association.^35^,^36^ Cognitive impairment in the absence of delirium or dementia has been less investigated. Evidence suggests that people admitted with moderate cognitive impairment are not at an increased risk of death in the short-term after admission,^2^,^36^ but no studies have longer-term follow-up. In contrast, mortality in people admitted with delirium superimposed on dementia has received extensive attention.^16^,^17^,^19^,^33^,^35^–^37^ Some studies have found no association with mortality at 1-year follow-up,^13^,^26^ whereas others found a significantly higher mortality.^19^,^35^,^36^ Explanations for this conflicting evidence include misclassification due to the difficulty of diagnosing delirium in the context of dementia^16^,^19^ and small sample sizes or recruitment from narrow clinical populations in some studies.^35^ However, an alternative explanation is that none of these studies examined whether mortality risks vary over time with CSD violating the proportional hazards assumption, meaning that averages are potentially misleading.

Functional decline has been identified as an important outcome in people with CSD,^18^,^20^,^36^ but there is no consensus on whether functional impairment explains worse mortality in people with CSD.^16^,^19^ Interpretation is complicated by CSD being an important cause of reduced functional ability. Hence, adjusting for ADL may simultaneously appropriately account for higher physical comorbidity and frailty in people with CSD and potentially over-adjust the effect of CSD on mortality. However, after adjustment for ADL, we found that CSD was still independently associated with increased mortality.

## Conclusion

One-third of medical inpatients have a CSD at admission and form a highly vulnerable population. Half will die in the 2 years after admission. Notably, after adjustment, having delirium alone is associated with higher early mortality during the 6 months after admission, whereas dementia (alone or with delirium superimposed) had no early increase in mortality but did have higher mortality after 3 months and unspecified cognitive impairment only after 6 months. The findings are suggestive that those with unspecified cognitive impairment may have early or undiagnosed dementia requiring follow-up after discharge to clarify diagnosis and optimize care. Additionally, this study does not support previous suggestions that delirium superimposed on dementia is associated with particularly poor prognosis, after adjusting for age and other covariates. This would be consistent with the role that established dementia plays as a predisposing factor for delirium onset, meaning that delirium in patients with dementia occurs with less severe physical disease precipitating factors and therefore that dementia rather than delirium dominates associations with mortality. In turn, in the absence of dementia, individuals require a larger acute insult in order to precipitate delirium, being more acutely unwell at the time of their delirium onset. Further research to explain the mortality patterns observed is required.

## Data availability

The data controller of the data analyzed is NHS Fife. Patientlevel data are available subject to standard information governance requirements for use of anonymized, unconsented NHS data (https://www.dundee.ac.uk/hic/).

## Figures and Tables

**Table 1:** Characteristics of people with CSD vs without CSD in terms of sex, age, residential status, deprivation, comorbidities, and functional status

	No CSDs	Any CSDs

All patients (N=6,724)	(N=4,344)	(N=2,380)
Sex		
Female (N=3,784)	2,375 (54.6)	1,409 (59.2)
Age (years), mean (SD)	77.6 (7.7)	82.1 (7.7)
65–69 (N=955)	788 (18.1)	167 (7)
70–74 (N=1,123)	867 (20.0)	256 (10.8)
75–79 (N=1,322)	902 (20.8)	421 (17.7)
80–84 (N=1,420)	871 (20.1)	549 (23.1)
85+ (N=1,904)	916 (21.1)	988 (41.5)
Residential status		
Care home (N=500)	75 (1.7)	425 (17.9)
SIMD^a^		
1, most deprived (N=1,376)	919 (21.2)	456 (19.2)
2 (N=1,789)	1,136 (26.1)	653 (27.4)
3 (N=1,548)	983 (22.6)	565 (23.7)
4 (N=1,032)	654 (15.1)	378 (15.9)
5, least deprived (N=979)	652 (15)	327 (13.7)
CCI groups^b^		
CCI 0 (N=1,629)	992 (22.8)	647 (27.2)
CCI 1 (N=1,728)	1,152 (26.5)	576 (24.2)
CCI 2–5 (N=2,733)	1,756 (40.4)	977 (41.1)
CCI 6+ (N=624)	449 (10.2)	180 (7.6)
No. of drugs^c^		
0 (N=389)	227 (5.2)	162 (6.8)
1–5 (N=1,725)	1,108 (25.5)	617 (25.9)
6–10 (N=2,650)	1,726 (39.7)	924 (38.8)
10+ (N=1,960)	1,283 (29.5)	677 (28.5)
ADL groups (N=4,846)^a^	(n=2,871)	(n=1,975)
Persistently low ADL (N=1,144)	314 (10.9)	830 (42.0)
Changed ADL (N=1,656)	886 (30.9)	770 (39.0)
Persistently high ADL (N=2,046)	1,671 (58.2)	375 (19.0)

**Notes:** All data are represented as n (%) except where indicated.

a Scottish Index of Multiple Deprivation divided into five quintiles.

b Charlson Comorbidity Index groups based on ICD10 codes in SMR01 data set.

c Number of drugs prescribed during the 84 days prior to admission.

d ADL based on current and 3 months prior to admission, 27% of which are missing.

**Abbreviations:** CSD, cognitive spectrum disorder; ADL, activity of daily living; CCI, Charlson Comorbidity Index; SIMD, Scottish Index of Multiple Deprivation.

**Table 2:** Results of the non-proportional hazards model showing hazard ratio estimates of associations with mortality for people with CSD

CSD	Time periods	Hazard ratio and 95% CI		
		Unadjusted model (a)	Adjusted model (b)	Adjusted + ADL (c)
Delirium alone vs no CSD	Up to 6 months	1.68 (1.48–1.90)	1.45 (1.28–1.65)	1.24 (1.08–1.42)
	6 months to 1 year	1.25 (0.97–1.62)	1.07 (0.82–1.38)	0.94 (0.72–1.22)
	1–2 years	1.69 (1.37–2.07)	1.44 (1.17–1.77)	1.27 (1.11–1.57)
Known dementia alone vs no CSD	Up to 3 months	1.39 (1.14–1.70)	1.03 (0.84–1.28)	0.86 (0.69–1.07)
	3 months to 2 years	2.39 (2.04–2.81)	1.85 (1.56–2.18)	1.55 (1.31–1.84)
Delirium and known dementia vs no CSD	Up to 3 months	1.68 (1.39–2.03)	1.18 (0.96–1.45)	0.98 (0.80–1.20)
	3 months to 2 years	2.48 (2.11–2.92)	1.80 (1.52–2.14)	1.49 (1.25–1.78)
Unspecified cognitive impairment vs no CSD	Up to 6 months	1.54 (1.23–1.93)	1.11 (0.87–1.40)	0.97 (0.77–1.21)
	6 months to 2 year	2.20 (1.72–2.82)	1.55 (1.21–1.99)	1.35 (1.05–1.74)

**Abbreviations:** CSD, cognitive spectrum disorder; ADL, activity of daily living.
